# Managing a gastrointestinal oncology practice in Japan during the COVID-19 pandemic: single institutional experience in The Cancer Institute Hospital of Japanese Foundation for Cancer Research

**DOI:** 10.1007/s10147-020-01806-7

**Published:** 2020-10-21

**Authors:** Daisuke Takahari, Eiji Shinozaki, Takeru Wakatsuki, Akira Ooki, Masato Ozaka, Takeshi Suzuki, Izuma Nakayama, Hiroki Osumi, Daisaku Kamiimabeppu, Taro Sato, Mariko Ogura, Mitsukuni Suenaga, Keisho Chin, Kensei Yamaguchi

**Affiliations:** 1 Department of Gastroenterology, The Cancer Institute Hospital of Japanese Foundation for Cancer Research, 3-8-31 Ariake, Koto-ku, Tokyo, 135-8550 Japan; 2 Gastroenterology Department, Kawasaki Municipal Hospital, Kawasaki, Japan

**Keywords:** Gastrointestinal oncology, COVID-19, Chemotherapy

## Abstract

Coronavirus disease 2019 (COVID-19) was declared to be a global pandemic by the World Health Organization on March 11, 2020. On April 7, 2020, a state of emergency was declared in Japan, as had been by other nations worldwide. This unprecedented crisis has profound implications for patients undergoing chemotherapy and for practicing healthcare professionals. Various reports have shown data indicating that cancer patients with COVID-19 have high morbidity and mortality rates. In order to reduce the use of medical resources to avoid the risk of COVID-19 infections in both cancer patients and health care providers, oncologists now have to draw the line for cancer treatments by maintaining their efficacy while avoiding severe adverse events. In this article, we outlined the decisions made regarding the practice of gastrointestinal oncology in our institution during the COVID pandemic.

## Introduction

A case of coronavirus disease 2019 (COVID-19), which is caused by the severe acute respiratory syndrome coronavirus 2 (SARS-CoV-2), was first reported in Wuhan City, China in December 2019. The World Health Organization (WHO) declared a global pandemic on March 11, 2020 [1]. As of May 20, 2020, there were 4,900,253 confirmed cases and 323,341 confirmed deaths worldwide [2]. The pandemic continues to spread over the globe. In Japan, as of 31 May, 2020, there were 16,851 cases of COVID-19 infection and 891 deaths [3]. On April 7, 2020, the Government of Japan declared a state of emergency under the Act on Special Measures for Pandemic Influenza and New Infectious Diseases Preparedness and Response. The areas where emergency measures were first implemented consisted of seven prefectures with the highest numbers of COVID-19 cases. However, on April 16, the emergency measures were expanded to include the entire country. The initial duration for the state of emergency was from April 7 to May 6, 2020, but then extended until May 31, 2020. Fortunately, it was lifted by May 25, but there is still a lot of caution to be taken. Even with the state of emergency, the government of Japan has worked to minimize its impact on social and economic functioning, and has not taken compulsory measures such as implementation of a "lockdown", which has been and is being implemented in other countries [4, 5]. For cancer patients, of course, hospital visits for treatment and assessments are not subject to restriction, but the visits themselves can be a risk of infection, especially for individuals who have risk factors of COVID-19 infection. The basic principle of preventing infection with SARS-CoV-2 is to reduce close contact with other people.

Although evidence remains insufficient, several reports have indicated that cancer patients have significantly increased severity and complications associated with COVID-19 infection [6–8]. A meta-analysis found that the odds ratio of severe complications was 2.29 [95% Confidence Interval (CI) 1.00–5.23] for patients with cancer [9]. The data suggest that patients with cancer more likely have severe disease leading to the need for intensive care and mechanical ventilation. And also, cancer patients with COVID-19 have high mortality rates [10–15]. The odds ratio of COVID-19 for death in patients with cancer and COVID-19 was reported to be 2.93 (95% CI 1.34–6.41, *p* = 0.006) [12]. For this reason, cancer societies around the world have issued guidelines for cancer patients and cancer treatment during the COVID-19 pandemic. The American Society of Clinical Oncology (ASCO) has issued a statement on general care for clinicians, the cancer care delivery team, and patients with cancer during the COVID-19 pandemic [16]; and the European Society of Medical Oncology (ESMO) also has issued a statement of encouragement for medical oncologists as follows: (1) ensure the continuum of care, (2) be prepared for new routines, (3) protect yourself to protect your patients, and (4) reinforce support to patients [17]. In addition, several recommendations, including United States (US), French, and Italian guidelines were published [18–20]. In Japan, three societies [the Japanese Cancer Society, the Japanese Society of Clinical Oncology, and the Japanese Society of Medical Oncology (JSMO)] recently established a joint working group that issued the publication “New Coronavirus Infection and Cancer Care (Q&A for Patients)” [21].

However, few specific recommendations on chemotherapy have been issued for patients with gastrointestinal (GI) cancers. Lou et al. recently proposed recommendations for minimizing risks to patients with GI cancers in the US [22].

The Cancer Institute Hospital of Japanese Foundation for Cancer Research is located in the center of Tokyo and is one of the largest cancer centers in Japan where provides approximately 20,000 GI cancer chemotherapy annually.

In this article, we present our clinical recommendations on how to modify the treatment but maintain effectiveness as much as possible for GI cancer patients receiving chemotherapies during the COVID-19 pandemic.

### Developing guidelines for patients with gastrointestinal cancers during the COVID-19 pandemic

Several guidelines from the US and Europe on chemotherapy during the COVID-19 pandemic have been published [16, 17]. The common strategies in these guidelines consist of the following: (1) balancing the benefits of treatment with risk of COVID-19 infection and exacerbation of cancer due to lack of treatment, and (2) reducing visits to the cancer center to reduce the risk of infection with SARS-CoV-2.

The risks of chemotherapy during the COVID-19 pandemic combined with the lack of guidance that takes into consideration the local circumstances in Japan have motivated us to develop a guidance for the use of chemotherapy in patients with GI cancers. The guidance was developed with the consensus of medical oncologists involved in GI cancer care in our institution (including 11 JSMO-certified oncology practitioners), who carefully considered whether the benefits of chemotherapy outweighed the risk of COVID-19 infection.

We examined the following questions:

1. What is the status of COVID-19 in Tokyo?
2. Does a particular therapy have enough evidence of clinical benefit to support it?
3. What are the considerations with regard to tumor burden and tumor growth rate?
4. Is the patient aged younger than 75 years with a performance status less than 2?
5. Are there any comorbidities such as hypertension, diabetic mellitus, cardiovascular/cerebrovascular disease, and chronic obstructive pulmonary disease that are relevant to the risk of COVID-19 severity?
6. How does the patient travel to our institution? Public or private transportation?
7. What is the patient's willingness to undergo treatment?

It was then discussed and finalized at each cancer multidisciplinary team conference with surgical and radiation oncologists, nurses, and pharmacists.

Table 1 shows the guidance on changes in treatments such as reducing the intensity of chemotherapy, delaying treatment, skipping cycles, or stopping according to the degree of severity of COVID-19 epidemic. Risk factors associated with COVID-19 are as follows: age (≥ 75 years), performance status ≥ 2, comorbidity (hypertension, diabetes mellitus, liver or renal or lung dysfunction). Low risk is defined as the presence of no risk factor. High risk is defined as the presence of one or more risk factors. Based on this concept, changes in treatments are considered for each type of GI cancer.

**Table 1:** Changes in treatments according to the degree of severity of COVID-19 epidemic

Level	Degree of epidemic	Low risk^a^	High risk^b^
1	Sporadic onset and mild increase of COVID-19	No change	Consider
2	Frequent onset and accelerating increase of COVID-19	Consider	Recommend
3	Spread of infection and overshoot of COVID-19	Recommend	Recommend

Changes in treatments: Reducing the intensity of chemotherapy, delaying treatment, skipping cycles, or stopping

Risk factors associated with COVID-19 are: age (≥ 75 years), performance status ≥ 2, comorbidity (hypertension, diabetes mellitus, liver or renal or lung dysfunction). These recommendations are based on single institutional experience

^a^Low risk is defined as the presence of no risk factor

^b^High risk is defined as the presence of one or more risk factors

### General considerations

Oncologists now have to draw the line for cancer treatments by maintaining their efficacy while avoiding severe adverse events and reducing the use of medical resources during the COVID-19 pandemic.

In accordance with ASCO recommendations [16], access to the institution is restricted to one entrance: all patients and visitors are screened there for fever, whether they have had contact with a patient with COVID-19, and screened for signs/symptoms (cough, dyspnea, and alterations in sense of smell or taste) of COVID-19. If the patient has had a fever greater than 37.5 °C during the previous 3 days or on the day of visit or any relevant signs/symptoms within 2 weeks, the patient will be transferred to a separate outpatient facility and undergo chest computed tomography (CT). If pneumonia is found or the attending physician deems it necessary after interviewing the patient, a polymerase chain reaction (PCR)-based SARS-CoV-2 assay of a patient specimen to detect the virus will be performed, and chemotherapy will not be administered on the same day, regardless of the patient’s signs/symptoms. Unlike influenza, the severity of COVID-19 is associated with acute respiratory distress syndrome (ARDS) rather than secondary bacterial pneumonia [23]. Therefore, the neutropenia resulting from chemotherapy might not pose a risk of mortality in COVID-19; however, in general, immunosuppression should be avoided.

Except for the regimens of docetaxel, cisplatin, 5-fluorouracil (DCF) used in esophageal squamous cell carcinoma (ESCC), 5-fluorouracil, leucovorin, irinotecan, oxaliplatin (FOLFOXIRI) used in colorectal cancer (CRC), and FOLFIRINOX in pancreatic cancer (PC), severe immunosuppression is not commonly associated with the chemotherapy regimens used in GI oncology. Under low risk of COVID-19 infection in the community, our routine clinical practice can usually follow established chemotherapy protocols. By contrast, for treating patients with high risk and/or during the pandemic, we must choose between reducing the intensity of chemotherapy (e.g., omission of the 5-FU bolus for CRC, replacement of infusional 5-FU by capecitabine or S-1 for ESCC, gastric cancer (GC), and CRC), delaying treatment, skipping cycles, or stopping (i.e., treatment maintenance phase and salvage chemotherapy with relatively few benefits). In addition, the empiric use of the prophylactic granulocyte colony-stimulating factor or antibiotics should be considered. We recommend the use of telemedicine for monitoring the side effects when treatment is delayed or chemotherapy cycles are skipped.

With regard to immunotherapy for GI cancers, immune-checkpoint inhibitor (ICI)s (nivolumab and pembrolizumab) can cause immune-related adverse events, including pneumonitis. We should accordingly weigh the potential clinical harm of immunotherapy in relation to its benefits, when considering its administration to a patient.

Furthermore, with regard to the use of anti-angiogenic agents (e.g., bevacizumab, ramucirumab, and aflibercept) for the treatment of GC and CRC, these agents are associated with a risk of arterial and venous thromboembolism, which have also been reported to occur at high rates in patients with COVID-19 [24, 25]. Although no evidence to avoid these anti-angiogenic agents during the pandemic is available, it is incumbent upon us to provide this information on COVID-19 to patients and healthcare providers. Moreover, hypertension is a frequent AE of antiangiogenic therapy, and antihypertensive drugs such as angiotensin II type 1 receptor blockers ARBs) or angiotensin-converting enzyme (ACE) inhibitors may affect expression of the ACE2, the receptor of SARS-Cov-2. So far, whether ACE inhibitors and ARBs worsen COVID-19-induced lung injury or not has been controversial [26, 27]. Thus, there is no need to discontinue it at this time, but caution should be exercised in the future.

### Esophageal cancer

#### Neoadjuvant chemotherapy

The standard neoadjuvant chemotherapy (NAC) used in Japan for clinical stage II/III ESCC is the combination of cisplatin plus 5-fluorouracil (CF) [28, 29]. A randomized controlled trial (JCOG9907) revealed its clinical benefit for curative resection and survival [28]. We should follow this principle of achieving curative resection and long-term survival, despite the COVID-19 pandemic. Several phase II studies [30, 31] found a clinical benefit for DCF in locally advanced ESCC. However, DCF, which is now being tested in the phase III trial JCOG1109, should not be recommended for NAC in clinical practice because of its hematological toxicity. In situations where surgery must be postponed or aborted because of various social situations, additional CF for NAC or changing treatment to definitive chemoradiation therapy should be considered.

#### Adjuvant chemotherapy

Adjuvant CF is indicated for patients in Japan with clinical stage II/III ESCC and with pathological nodal metastatic disease who received surgery without NAC [32]. However, the survival benefit of adjuvant CF was not definitive in the JCOG9204 trial [32]. Accordingly, adjuvant CF should be omitted during the COVID-19 pandemic.

#### Palliative chemotherapy

We also avoid DCF in the palliative setting because of the reasons listed in the previous section on ‘Neoadjuvant chemotherapy’. With regard to palliative chemotherapy in the patients with high risk of COVID-19 infection, reduction in treatment intensity should be considered. Best supportive care can be an option, depending on the situation.

For first-line systemic chemotherapy, we might consider changing from CF to oral S-1 monotherapy. For second-line chemotherapy, nivolumab, which showed survival benefit in the ATTRACTION-3 trial [33], should be considered for administration with longer intervals between each administration. In addition, for patients receiving nivolumab for longer than 12 months, pausing its administration could be considered [10, 16]. Taxanes such as paclitaxel or docetaxel can sometimes be candidates for third-line chemotherapy. Intervals between administration of these agents should also be longer, as the situation demands, because they sometimes cause severe neutropenia. Prophylactic granulocyte-colony stimulating factor should also be considered.

#### Concurrent radiotherapy (RT)

A total radiation dose of 60 Gy is often used for chemoradiotherapy in stage I or advanced ESCC [34, 35]. However, according to the RTOG regimen, 50.4 Gy of RT is generally used worldwide [36]. According to the RADS (Remote, Avoid, Defer, Shorten) principle [37], we have adopted 50 Gy/25 fractions (Fr) instead of 60 Gy/30 Fr to reduce the immunosuppressive effect of RT during the COVID-19 pandemic.

Table 2 lists the principles of chemotherapy for ESCC according to each risk group in the COVID-19 pandemic.

**Table 2:** Principles of chemotherapy for esophageal cancer according to each risk group in the COVID-19 pandemic

Treatment settings	Low risk^a^	High risk^b^
Neoadjuvant	CF	CF with dose reduction or 5-fluorouracil monotherapy
Adjuvant	Unrecommended	Unrecommended
Definitive chemoradiation	RT (50 Gy/25fr) plus CF	RT (50 Gy/25fr) plus either CF with dose reduction or 5-fluorouracil monotherapy
Palliative (1st line)	CF	CF with dose reduction or 5-fluorouracil or S-1 monotherapy or BSC
Palliative (2nd line)	Nivolumab (biweekly)	Nivolumab (every 3 or more weeks) or BSC
Palliative (3rd line)	Paclitaxel or Docetaxel or BSC	Paclitaxel with less frequent dosing intervals or Docetaxel with G-CSF or BSC

Risk factors associated with COVID-19 are: age (≥ 75 years), performance status ≥ 2, comorbidity (hypertension, diabetes mellitus, liver or renal or lung dysfunction). These recommendations are based on single institutional experience

*CF* cisplatin plus 5-fluorouracil; *RT* radiotherapy; *BSC* best supportive care; *G-CSF* granulocyte-colony stimulating factor

^a^Low risk is defined as the presence of no risk factor

^b^High risk is defined as the presence of one or more risk factors

### Gastric cancer

#### Neoadjuvant chemotherapy

NAC is not the standard treatment for resectable GC in Japan [38]. Thus, based on the results of the JCOG0405 trial [39], we only recommend NAC for resectable GC with bulky and/or para-aortic lymph node metastases for patients with low risk of COVID-19 infection.

#### Adjuvant chemotherapy

Gastrectomy followed by adjuvant chemotherapy is the standard of care for patients with resectable GC in Japan [38]. The Adjuvant chemotherapy trial of S-1 for gastric cancer (ACTS-GC) trial verified the efficacy of S-1 monotherapy as adjuvant chemotherapy for Stage II or III GC [40, 41]. The Japan Clinical Cancer Research Organization (JACCRO)-GC 07 trial recently demonstrated that compared to S-1 monotherapy, the addition of docetaxel to S-1 (DS) significantly prolonged recurrence-free survival (RFS) for stage III GC [42]. Based on the results of these trials, S-1 monotherapy for Stage II and DS for Stage III GC patients is the current standard adjuvant chemotherapy [38]. Although the interim analysis of the JACCRO-GC 07 trial showed that the superiority of DS compared with S-1 monotherapy in terms of 3-year RFS, the improvement in overall survival (OS) remains unclear. In addition, severe (grade 3/4) neutropenia was observed twice as often in the DS (38.1%) than in the S-1 (16.1%) patients [42].

Furthermore, the ACTS-GC trial did not prove that S-1 was effective as adjuvant chemotherapy for patients aged older than 80 years [3]. Taken together, regarding indications of adjuvant chemotherapy, S-1 alone for Stage III GC patients with high risk of COVID-19 infection is recommended and adjuvant chemotherapy for elderly patients (> 80 years) is not recommended. Patients receiving ongoing adjuvant chemotherapy and without severe hematologic toxicity before the onset of the COVID-19 pandemic should continue receiving that same treatment during the pandemic; however, dose reduction or stopping should be considered for patients with high risk of COVID-19 infection.

#### Palliative chemotherapy

In principle, we recommend continuing of the administration of the same standard chemotherapy to patients with low risk of COVID-19 infection, even during the COVID-19 pandemic. However, reducing the frequency of hospital visits by modifying treatment schedules (such as reducing the intensity of chemotherapy, delaying treatment or skipping cycles) for patients with high risk of COVID-19 infection may be considered.

As first-line chemotherapy, for patients with high tumor burden or tumor-related signs/symptoms or low risk of COVID-19 infection, combination of fluoropyrimidine and oxaliplatin (if HER2 is positive, plus trastuzumab) is recommended as a standard regimen [38]. For patients with cytology-positive (CY1) tumors who were considered for conversion surgery, the treatment intensity should be maintained. On the other hand, for patients with low tumor burden or high risk of COVID-19 infection, non-intensive monotherapy [S-1 plus oxaliplatin (SOX)] with reduced dose of oxaliplatin, S-1 monotherapy, or 5-FU/leucovorin) is an alternative regimen. Prescription for two courses at once will be allowed for patients who received S-1 monotherapy as maintenance therapy to reduce the frequency of their hospital visits.

As second line therapy, ramucirumab plus paclitaxel /nanoparticle albumin-bound (nab)-paclitaxel is the standard of care in Japan [38, 43, 44]. We propose skipping the administration of paclitaxel /nab-paclitaxel at day 8 to avoid neutropenia and to reduce the frequency of hospital visits. A previously published study has indicated that biweekly administration of ramucirumab plus paclitaxel did not compromise efficacy [45].

As third-line therapy, the subgroup analysis of Japanese patients in the ATTRACTION-2 trial reported a very low frequency of immune-related pneumonitis (*n* = 1, 0.7% of 152 patients) [46]. Therefore, nivolumab is recommended in third or later line, but also consider prolonging the interval between administration (3–6 weeks) for responders. Irinotecan and trifluridine/tipiracil are also third-line or later treatment options for GC [47, 48]. However, these two agents show a relatively high risk of severe hematologic toxicity (> 30%) [47, 48]. Thus, the treatment modification (reducing the intensity of chemotherapy, delaying treatment, skipping cycles, or stopping) should be considered, especially for patients with high-risk factors of COVID-19 infection.

Because it is associated with less hematologic toxicity than irinotecan and trifluridine/tipiracil, ramucirumab monotherapy could also be a treatment option for patients with high risk of COVID-19 infection [49, 50].

Table 3 lists the principles of chemotherapy for GC according to each risk group in the COVID-19 pandemic.

**Table 3:** Principles of chemotherapy for gastric cancer according to each risk group in the COVID-19 pandemic

Treatment settings	Low risk^a^	High risk^b^
Neoadjuvant	SOX (for only bulky N)	Unrecommended
Adjuvant	Stage II: S-1 Stage III: DS	StageII/III: 75–79 y/o: S-1 with reduced dose, ≥ 80 y/o: surgery alone
Palliative (1st line)	SOX ± Tmab^c^	SOX ± Tmab^c^ with delay interval of reduced dose of SOX or S-1 ± Tmab^c^ with reduced dose of S-1
Palliative (2nd line)	PTX/nabPTX ± RAM (bi-weekly)	PTX/nabPTX ± RAM (with delay interval of bi-weekly) or PTX/nabPTX (with delay interval of bi-weekly) or BSC
Palliative (3rd line or later)	Nivolumab or FTD/TPI or IRI	Nivolumab (with delay interval) or FTD/TPI with delay interval or reduced dose or IRI with delay interval of reduced dose or RAM alone or BSC

Risk factors associated with COVID-19 are: age (≥ 75 years), performance status ≥ 2, comorbidity (hypertension, diabetes mellitus, liver or renal or lung dysfunction). These recommendations are based on single institutional experience

*SOX* S-1 plus oxaliplatin; *DS* docetaxel plus S-1; *Tmab* trastuzumab; *PTX* paclitaxel; *RAM* ramucirumab; *BSC* best supportive care; *FTD/TPI* trifluridine/tipiracil; *IRI* Irinotecan

^a^Low risk is defined as the presence of no risk factor

^b^High risk is defined as the presence of one or more risk factors

^c^HER2 positive case: + Tmab

### Colorectal cancer

#### Neoadjuvant chemotherapy (including chemoradiotherapy)

For patients with low risk of COVID-19 infection, we should start and continue standard neoadjuvant chemotherapy (chemoradiotherapy), if possible. However, short-course radiotherapy (5 × 5 Gy) followed by delayed surgery may be the best option, except for T4 rectal cancer. It has been demonstrated that the pathological complete response rate was significantly higher in patients whose surgery was delayed than in patients who underwent immediate surgery, although there were no differences in rates of preservation of sphincters and negative margins of resected tumors between two groups [51]. Neoadjuvant chemotherapy is not recommended for patients with high risk of COVID-19 infection.

#### Adjuvant chemotherapy

For patients with low risk of COVID-19 infection, we should start and continue standard adjuvant chemotherapy if possible (T3 and N1: capecitabine plus oxaliplatin (CapeOx) 3–6 months, T4 and/or N2: CapeOX 6 months) [52]. However, adjuvant chemotherapy that reduces the intensity and shortens the duration of chemotherapy is an alternative option. Treatment should be performed at a local hospital near where a patient resides if traveling to the cancer center is too difficult.

For patients with high risk of COVID-19 infection, adjuvant chemotherapy is not recommended. Fluoropyrimidine monotherapy is the alternative option of the adjuvant chemotherapy.

For patients with microsatellite instability high (MSI-H)/deficient mismatch repair (dMMR), patients receiving adjuvant chemotherapy for stages II and III MSI-H/dMMR CRC have a lower recurrence rate than MSS/pMMR CRC patients, and also a favorable prognosis [53].

#### Palliative chemotherapy

We should start and continue standard chemotherapy according to the National Comprehensive Cancer Network (NCCN)/ESMO/Japanese Society for Cancer of the Colon and Rectum (JSCCR) guidelines for patients with metastatic CRC and high tumor burden or who expect to undergo conversion surgery, [54–56] if possible. Furthermore, non-intensive chemotherapies such as fluoropyrimidines (5-FU, capecitabine and S-1) and bevacizumab, fluoropyrimidine monotherapy, or a stop-and-go strategy are alternative options.

For patients with a low tumor burden or high-risk factors, non-intensive chemotherapy may be desirable. Furthermore, delaying treatment and treating at the patient’s local hospital should be considered if traveling to the cancer center is too difficult.

We consider using pembrolizumab for patients with MSI-H/dMMR regardless of risk factors associated with severe COVID-19 [57], although there are several controversial issues, such as the differential diagnosis between COVID-19- related lung injury and ICI-associated immune-related adverse events and the risk of worsening lung injury by activating the immune response.

Table 4 lists the principles of chemotherapy for CRC according to each risk group in the COVID-19 pandemic.

**Table 4:** Principles of chemotherapy for colorectal cancer according to each risk group in the COVID-19 pandemic

Treatment setting	High tumor burden/ Low risk^a^	Low tumor burden/high risk^b^	MSI-H/dMMR
Neoadjuvant	Standard chemotherapy/chemoradiotherapy (alternative: short course RT)	Unrecommended	Depends on tumor burden and risk factors
Adjuvant	Standard chemotherapy, 3–6 months (alternative: replacement of 5-FU to capecitabine, option: FU monotherapy, treatment at the local hospitals)	Unrecommended (option: FU monotherapy)	Unrecommended
Palliative	Standard chemotherapy (alternative: replacement of 5-FU to capecitabine, option: non-intensive chemotherapy)	Non-intensive chemotherapy (option: treatment delay, treatment at the local hospitals)	Standard chemotherapy or Pembrolizumab

Risk factors associated with COVID-19 are: age (≥ 75 years), performance status ≥ 2, comorbidity (hypertension, diabetes mellitus, liver or renal or lung dysfunction). These recommendations are based on single institutional experience

*MSI-H* microsatellite instability high, *dMMR* deficient mismatch repair, *RT* radiotherapy, FU montherapy: capecitabine, S-1, tegafur-uracil/Leucovorin for 6 months, Non-intensive chemotherapy: Fluoropyrimidine and bevacizumab, Fluoropyrimidine monotherapy or a stop-and-go strategy

^a^Low risk is defined as the presence of no risk factor

^b^High risk is defined as the presence of one or more risk factors

## Biliary-pancreatic cancer

### Pancreatic cancer

#### Adjuvant chemotherapy

The survival benefit of S-1 monotherapy as adjuvant chemotherapy for PC has been shown by the JASPAC-01 study [58]. The recent results of the Prep-02/JSAP05 trial have revealed the survival benefit of gemcitabine plus S-1 (GS) as neoadjuvant chemotherapy for resectable PC [59]. Based on these results, NAC with GS and postoperative adjuvant chemotherapy with S-1 have become the standard of care for patients with resectable PC in Japan.

Considering the poor outcome of treating PC with surgery alone and the significant survival-prolonging effect of adjuvant (neoadjuvant) chemotherapy, NAC with GS and postoperative adjuvant chemotherapy with S-1 are recommended even during the COVID-19 pandemic. The initiation of adjuvant chemotherapy can be delayed until 12 weeks after surgery if the patient does not recover sufficiently after surgery. FOLFIRINOX is not recommended as postoperative adjuvant chemotherapy, because severe neutropenia has been reported, and it is not covered by insurance in Japan.

#### Palliative chemotherapy

There is no indication for discontinuing or changing the current chemotherapy in patients with low risk of COVID-19 infection. However, if a new treatment is planned, gemcitabine plus nab-paclitaxel, which has a relatively low incidence of hematologic toxicity, is recommended [60]. FOLFOX may be considered in cases in which platinum is preferred, such as in patients with breast cancer gene (BRCA) mutations [61].

For elderly patients or those with high risk of COVID-19 infection, changes in treatment intervals or a change to S-1 monotherapy [62, 63] may be considered.

For patients undergoing gemcitabine plus nab-paclitaxel, the deletion of day 8 of the standard regimen, namely, providing infusions of both drugs on days 1 and 15 of each 28-day cycle, should be considered to avoid neutropenia and to reduce the frequency of hospital visits. Some studies have reported that this approach improves patient tolerance without significantly compromising efficacy [64, 65].

Concerning second-line treatment, we need to evaluate the indications for the treatment carefully for not only new patients but also patients currently undergoing treatment. Best supportive care can be an option as the situation demands.

### Biliary tract cancer

#### Adjuvant chemotherapy

In Japan, there is no evidence of improved survival for biliary tract cancer patients treated with adjuvant or neoadjuvant chemotherapy. Adjuvant/neoadjuvant chemotherapy should not be administered to biliary tract cancer patients during the COVID-19 pandemic.

#### Palliative chemotherapy

There is no indication for discontinuing or changing current chemotherapy in patients with low risk of COVID-19 infections. Gemcitabine plus cisplatin is the standard of care for patients with unresectable biliary tract cancer [66]. Intervals between chemotherapy cycles, such as a biweekly regimen, should be lengthened as the situation demands because gemcitabine plus cisplatin sometimes causes severe neutropenia.

Table 5 lists the principles of chemotherapy used for biliary-pancreatic cancer according to each risk group in the COVID-19 pandemic.

**Table 5:** Principles of chemotherapy for biliary-pancreatic cancer according to each risk group in the COVID-19 pandemic

Treatment settings	Low risk^a^	High risk^b^
Pancreatic cancer		
Neoadjuvant	GS	GS
Adjuvant	S-1	S-1
Palliative (1st line)	GnP	GnP or S-1 monotherapy or BSC
Palliative (2nd line)	S-1	BSC
Biliary tract cancer		
Neoadjuvant	Unrecommended	Unrecommended
Adjuvant	Unrecommended	Unrecommended
Palliative (1st line)	GC	Gem monotherapy or S-1 or BSC
Palliative (2nd line)	S-1	Unrecommended

Risk factors associated with COVID-19 are: age (≥ 75 years), performance status ≥ 2, comorbidity (hypertension, diabetes mellitus, liver or renal or lung dysfunction). These recommendations are based on single institutional experience

*GS* gemcitabine plus S-1; *GnP* gemcitabine plus nab-paclitaxel; *BSC* best supportive care; *GC* gemcitabine plus Cisplatin

^a^Low risk is defined as the presence of no risk factor

^b^High risk is defined as the presence of one or more risk factors

## Discussion

In this article, we outlined the recommendations for making decisions on chemotherapies for GI cancers during the COVID pandemic. There are several recommendations and guidelines already published worldwide (16–21). However, few specific recommendations on chemotherapy have been issued for patients with GI cancers. Moreover, practices differ in many aspects in GI cancers between US/Europe and Japan (e.g., oral agent such as S-1 are widely used). And the guidelines from Japan are general and do not specifically address how to modify the treatment but maintain effectiveness as this article described. In this respect, we believe that our guidance is based on actual clinical practice of GI oncology in Japan and would be useful not only to cancer center institutions but also to general hospitals.

Now, providing normal medical care is also being somewhat limited because of COVID-19. The standard approach for GI cancer, early detection and early treatment, such as that of endoscopic screening and endoscopic submucosal dissection (ESD), respectively, which have been very prevalent in Japanese society, is in a crisis. In addition, compared to other countries, a higher number of Japanese patients have received subsequent chemotherapy after disease progression [67]. This strategy of early detection and treatment and higher rates of subsequent implementation of chemotherapy might have contributed to longer survival in Japanese patients than the survival of other populations. However, retaining a similar treatment strategy after the onset of the COVID-19 pandemic might be difficult.

Despite these difficulties, we must continue to administer chemotherapy to the patients who need it. The most important goal of treatment was to complete the protocol without reducing the intensity of treatment, especially if cure is desired. In the palliative setting, however (in fact, the majority of cases are in the palliative setting), minimizing the risk of developing severe COVID-19 that is associated with chemotherapy and maximizing survival and symptom relief are important. In order to achieve these important goals, the duration of treatments should be modified; and dose reduction, treatments with reduced toxicity, change to oral agents such as S-1 that require reduced visits to the cancer center, and empirical use of granulocyte-stimulating factor should be considered.

Along with protecting the patient from infection, infection of healthcare providers must be avoided. If healthcare providers are infected, and the medical supply system is not maintained, anticancer treatments must be reduced. For example, our department has created a business continuity plan that limits the number of treatments based on proportion of staff that is absent because of COVID-19 infection. Such preparations are important for any medical institution.

With regard to ongoing clinical trials, with the considerations for protecting patients and the institutional standards for Good Clinical Practice (GCP) during the COVID-19 pandemic state, recruitment of new patients was avoided as own consensus in our institution, and the safety for the current participants should be prioritized. Guidance is also provided by the Food and Drug Administration [68] and referring to it is recommended.

The limitation of this guidance is that our recommendations are not comprehensively evidence-based and are largely based on expert opinion. We have just started using it in our institution and its feasibility should be confirmed. Currently, several issues on COVID-19 and cancer chemotherapy have been published in addition to ours, but all of them remain controversial. The accumulation of evidence-based data should lead to the establishment of optimal treatment policies. As effective treatments and vaccines for COVID-19 are developed, this guidance may need updating, and there will be additional new issues to consider, such as the risks associated with concomitant use of vaccine against COVID-19 and ICI therapy. Staying updated with new information for the treatments of GI cancers is important.

In summary, we in the field of GI oncology recommend considering modifications to conventional chemotherapy that are based on the prevalence of COVID-19, treatment goals and patients’ vulnerabilities. Even if the pandemic is successfully suppressed, subsequent pandemics will inevitably occur. These recommendations should, therefore, be useful in the post-Corona era as well.

## References

[1] World Health Organization (2020) Coronavirus Disease (COVID-19) Outbreak; 2020. https://www.who.int/emergencies/diseases/novel- coronavirus-2019. Accessed 18 Mar 2020.

[2] Johns Hopkins University (2020) COVID-19 Dashboard by the Center for Systems Science and Engineering at Johns Hopkins University. https://gisanddata.maps.arcgis.com/apps/opsdashboard/index. Accessed 20 May 2020.

[3] Ministry of Health, Labor and Health Care Japan (2020) https://www.mhlw.go.jp/stf/seisakunitsuite/bunya/0000164708_00001.html#kokunaihassei. Accessed 20 May 2020.

[4] Ministry of Health, Labor and Health Care Japan (2020) https://www.mhlw.go.jp/content/10900000/000620733pdf. Accessed 18 Mar 2020.

[5] Ministry of Health, Labor and Health Care Japan (2020) https://www.mhlw.go.jp/content/10900000/000624195pdf. Accessed 18 Mar 2020.

[6] Liang W, Guan W, Chen R (2020). Cancer patients in SARS-CoV-2 infection: a nationwide analysis in China. Lancet Oncol.

[7] Xia Y, Jin R, Zhao J (2020). Risk of COVID-19 for patients with cancer. Lancet Oncol.

[8] Guan WJ, Liang WH, Zhao Y (2020). Comorbidity and its impact on 1590 patients with Covid-19 in China: a nationwide analysis. Eur Respir J.

[9] Wang B, Li R, Lu Z (2020). Does comorbidity increase the risk of patients with COVID-19: evidence from meta-analysis. Aging (Albany NY).

[10] World Health Organization (2020). Report of the WHO-China Joint Mission on Coronavirus Disease 2019 (COVID-19).

[11] Dai M, Liu D, Liu M (2020). Patients with cancer appear more vulnerable to SARS-COV-2: a multicenter study during the COVID-19 outbreak. Cancer Discov.

[12] Deng G, Yin M, Chen X (2020). Clinical determinants for fatality of 44,672 patients with COVID-19. Crit Care.

[13] Miyashita H, Mikami T, Chopra N (2020). Do patients with cancer have a poorer prognosis of COVID-19? an experience in New York city. Ann Oncol.

[14] Onder G, Rezza G, Brusaferro S (2020) Case-fatality rate and characteristics of patients dying in relation to COVID-19 in Italy. JAMA March 23. Online ahead of print 10.1001/jama.2020.4683 32203977

[15] Mehta V, Goel S, Kabarriti R (2020). Case fatality rate of cancer patients with COVID-19 in a New York hospital system. Cancer Discov.

[16] American Society of Clinical Oncology (2020) COVID-19 Patient Care Information. https://www.asco.org/asco-coronavirus-information/care-individuals-cancer-during-covid-19. Accessed 7 May 2020.

[17] European Society of Medical Oncology (2020) COVID-19: supporting oncology professionals. https://www.esmo.org/covid-19-and-cancer/supporting-oncology-professionals. Accessed 7 May 2020.

[18] Schrag D, Hershman DL, Basch E (2020). Oncology practice during the COVID-19 pandemic. JAMA.

[19] You B, Ravaud A, Canivet A (2020). The official French guidelines to protect patients with cancer against SARS-CoV-2 infection. Lancet Oncol.

[20] Pinto C, Cagnazzo C (2020). Indications regarding the management of interventional clinical trials with drugs during the current COVID-19 emergency in Italy. ESMO Open.

[21] Japanese Cancer Society (2020) The Japanese Society of Clinical Oncology, and the Japanese Society of Medical Oncology. https://www.jca.gr.jp/public/c_q_and_a.html Accessed 15 May 2020.

[22] Lou E, Beg S, Bergsland E (2020). Modifying practices in GI oncology in the face of COVID-19: recommendations from expert oncologists on minimizing patient risk. JCO Oncol Pract.

[23] Morens DM, Taubenberger JK, Fauci AS (2008). Predominant role of bacterial pneumonia as a cause of death in pandemic influenza: implications for pandemic influenza preparedness. J Infect Dis.

[24] Lodigiani C, Iapichino G, Carenzo L (2020). Venous and arterial thromboembolic complications in COVID-19 patients admitted to an academic hospital in Milan, Italy. Thromb Res.

[25] Mehra MR, Desai SS, Kuy S et al (2020) Cardiovascular disease, drug therapy, and mortality in Covid-19. N Engl J Med 382(25):e102. 10.1056/NEJMoa2007621 [Retraction in: N Engl J Med 2020] 10.1056/NEJMoa2007621 PMC7206931 32356626

[26] Vaduganathan M, Vardeny O, Michel T (2020). Renin-angiotensin-aldosterone system inhibitors in patients with Covid-19. N Engl J Med.

[27] Li J, Wang X, Chen J (2020). Association of renin-angiotensin system inhibitors with severity or risk of death in patients with hypertension hospitalized for Coronavirus Disease 2019 (COVID-19) infection in Wuhan, China. JAMA Cardiol.

[28] Ando N, Kato H, Igaki H (2012). A Randomized trial comparing postoperative adjuvant chemotherapy with cisplatin and 5-fluorouracil versus preoperative chemotherapy for localized advanced squamous cell carcinoma of the thoracic esophagus (JCOG9907). Ann Surg Oncol.

[29] Kitagawa Y, Uno T, Oyama T (2019). Esophageal cancer practice guidelines 2017 edited by the Japan Esophageal Society: part 1. Esophagus.

[30] Yokota T, Hatooka S, Ura T (2011). Docetaxel plus 5-fluorouracil and cisplatin (DCF) induction chemotherapy for locally advanced borderline-resectable T4 esophageal cancer. Anticancer Res.

[31] Yokota T, Kato K, Hamamoto Y (2016). Phase II study of chemoselection with docetaxel plus cisplatin and 5-fluorouracil induction chemotherapy and subsequent conversion surgery for locally advanced unresectable oesophageal cancer. Br J Cancer.

[32] Ando N, Iizuka T, Ide H (2003). Surgery plus chemotherapy compared with surgery alone for localized squamous cell carcinoma of the thoracic esophagus: a Japan Clinical Oncology Group Study—JCOG9204. J Clin Oncol.

[33] Kato K, Cho BC, Takahashi M (2019). Nivolumab versus chemotherapy in patients with advanced oesophageal squamous cell carcinoma refractory or intolerant to previous chemotherapy (ATTRACTION-3): a multicentre, randomised, open-label, phase 3 trial. Lancet Oncol.

[34] Kato H, Sato A, Fukuda H (2009). A phase II trial of chemoradiotherapy for stage i esophageal squamous cell carcinoma: Japan Clinical Oncology Group Study (JCOG9708). Jpn J Clin Oncol.

[35] Shinoda M, Ando N, Kato K (2015). Randomized study of low-dose versus standard-dose chemoradiotherapy for unresectable esophageal squamous cell carcinoma (JCOG0303). Cancer Sci.

[36] Minsky BD, Pajak TF, Ginsberg RJ (2002). INT 0123 (Radiation Therapy Oncology Group 94–05) phase III trial of combined-modality therapy for esophageal cancer: high-dose versus standard-dose radiation therapy. J Clin Oncol.

[37] Zaorsky NG, Yu JB, McBride SM (2020). Prostate cancer radiotherapy recommendations in response to COVID-19. Adv Radiat Oncol.

[38] Japanese Gastric Cancer Association (2020). Japanese gastric cancer treatment guidelines 2018 (5th edition). Gastric Cancer.

[39] Tsuburaya A, Mizusawa J, Tanaka Y (2014). Neoadjuvant chemotherapy with S-1 and cisplatin followed by D2 gastrectomy with para-aortic lymph node dissection for gastric cancer with extensive lymph node metastasis. Br J Surg.

[40] Sakuramoto S, Sasako M, Yamaguchi T (2007). Adjuvant chemotherapy for gastric cancer with S-1, an oral fluoropyrimidine. N Engl J Med.

[41] Sasako M, Sakuramoto S, Katai H (2011). Five-year outcomes of a randomized phase III trial comparing adjuvant chemotherapy with S-1 versus surgery alone in stage II or III gastric cancer. J Clin Oncol.

[42] Yoshida K, Kodera Y, Kochi M (2019). Addition of docetaxel to oral fluoropyrimidine improves efficacy in patient with Stage III gastric cancer: Interim analysis of JACCRO GC-07, a randomized controlled trial. J Clin Oncol.

[43] Wilke H, Muro K, Van Cutsem E (2014). Ramucirumab plus paclitaxel versus placebo plus paclitaxel in patients with previously treated advanced gastric or gastro-oesophageal junction adenocarcinoma (RAINBOW): a double-blind, randomised phase 3 trial. Lancet Oncol.

[44] Bando H, Shimodaira H, Fujitani K (2018). A phase II study of nab-paclitaxel in combination with ramucirumab in patients with previously treated advanced gastric cancer. Eur J Cancer.

[45] Rogers JE, Xiao L, Amlashi FG (2019). Ramucirumab and paclitaxel administered every 2 weeks (mRAINBOW Regimen) in advanced gastroesophageal adenocarcinoma. Oncology.

[46] Kato K, Satoh T, Muro K (2019). A subanalysis of Japanese patients in a randomized, double-blind, placebo-controlled, phase 3 trial of nivolumab for patients with advanced gastric or gastro-esophageal junction cancer refractory to, or intolerant of, at least two previous chemotherapy regimens (ONO-4538-12, ATTRACTION-2). Gastric Cancer.

[47] Shitara K, Doi T, Dvorkin M (2018). Trifluridine/tipiracil versus placebo in patients with heavily pretreated metastatic gastric cancer (TAGS): a randomised, double-blind, placebo-controlled, phase 3 trial. Lancet Oncol.

[48] Makiyama A, Arimizu K, Hirano G (2018). Irinotecan monotherapy as third-line or later treatment in advanced gastric cancer. Gastric Cancer.

[49] Fuchs CS, Tomasek J, Yong CJ (2014). Ramucirumab monotherapy for previously treated advanced gastric or gastro-oesophageal junction adenocarcinoma (REGARD): an international, randomised, multicentre, placebo-controlled, phase 3 trial. Lancet.

[50] Murahashi S, Takahari D, Wakatsuki T (2018). A retrospective analysis of ramucirumab monotherapy in previously treated Japanese patients with advanced or metastatic gastric adenocarcinoma. Int J Clin Oncol.

[51] Bujko K, Partycki M, Pietrzak L (2014). Neoadjuvant radiotherapy (5 × 5 Gy): immediate versus delayed surgery. Recent Results Cancer Res.

[52] Grothey A, Sobrero AF, Shields AF (2018). Duration of adjuvant chemotherapy for stage III colon cancer. N Engl J Med.

[53] Des Guetz G, Schischmanoff O, Nicolas P (2009). Does microsatellite instability predict the efficacy of adjuvant chemotherapy in colorectal cancer? A systematic review with meta-analysis. Eur J Cancer.

[54] Benson AB, Venook AP, Al-Hawary MM (2018). NCCN guidelines insights: colon cancer, version 2.2018. J Natl Compr Canc Netw.

[55] Yoshino T, Arnold D, Taniguchi H (2018). Pan-Asian adapted ESMO consensus guidelines for the management of patients with metastatic colorectal cancer: a JSMO-ESMO initiative endorsed by CSCO, KACO, MOS, SSO and TOS. Ann Oncol.

[56] Hashiguchi Y, Muro K, Saito Y (2020). Japanese Society for Cancer of the Colon and Rectum (JSCCR) guidelines 2019 for the treatment of colorectal cancer. Int J Clin Oncol.

[57] Overman MJ, McDermott R, Leach JL (2017). Nivolumab in patients with metastatic DNA mismatch repair-deficient or microsatellite instability-high colorectal cancer (CheckMate 142): an open-label, multicentre, phase 2 study. Lancet Oncol.

[58] Uesaka K, Boku N, Fukutomi A (2016). Adjuvant chemotherapy of S-1 versus gemcitabine for resected pancreatic cancer: a phase 3, open-label, randomised, non-inferiority trial (JASPAC 01). Lancet.

[59] Motoi F, Kosuge T, Ueno H (2019). Randomized phase II/III trial of neoadjuvantchemotherapy with gemcitabine and S-1 versus upfront surgery for resectable pancreatic cancer( Prep-02/JSAP- 05). Jpn J Clin Oncol.

[60] Von Hoff DD, Ervin T, Arena FP (2013). Increased survival in pancreatic cancer with nab-paclitaxel plus gemcitabine. N Engl J Med.

[61] Fogelman D, Sugar EA, Oliver G (2015). Family history as a marker of platinum sensitivity in pancreatic adenocarcinoma. Cancer Chemother Pharmacol.

[62] Todaka A, Fukutomi A, Boku N (2010). S-1 monotherapy as second-line treatment for advanced pancreatic cancer after gemcitabine failure. Jpn J Clin Oncol.

[63] Sudo K, Yamaguchi T, Nakamura K (2011). Phase II study of S-1 in patients with gemcitabine-resistant advanced pancreatic cancer. Cancer Chemother Pharmacol.

[64] Ahn DH, Krishna K, Blazer M (2017). A modified regimen of biweekly gemcitabine and nab-paclitaxel in patients with metastatic pancreatic cancer is both tolerable and effective: a retrospective analysis. Ther Adv Med Oncol.

[65] Kokkali S, Tripodaki E-S, Drizou M (2018). Biweekly gemcitabine/nab-paclitaxel as first-line treatment for advanced pancreatic cancer. Vivo.

[66] Valle J, Wasan H, Palmer DH (2010). Cisplatin plus gemcitabine versus gemcitabine for biliary tract cancer. N Engl J Med.

[67] Sawaki A, Yamada Y, Yamaguchi K (2018). Regional differences in advanced gastric cancer: exploratory analyses of the AVAGAST placebo arm. Gastric Cancer.

[68] The Food and Drug Administration (2020) FDA Guidance on Conduct of Clinical Trials of Medical Products during COVID-19 Public Health Emergency. https://www.fda.gov/regulatory-information/search-fda-guidance-documents/fda-guidance-conduct-clinical-trials-medical-products-during-covid-19-public-health-emergency. Accessed 20 May 2020.

